# Insoluble-Bound Polyphenols Released from Guarana Powder: Inhibition of Alpha-Glucosidase and Proanthocyanidin Profile

**DOI:** 10.3390/molecules25030679

**Published:** 2020-02-05

**Authors:** Ana Clara da Costa Pinaffi, Geni Rodrigues Sampaio, Maiara Jurema Soares, Fereidoon Shahidi, Adriano Costa de Camargo, Elizabeth A. F. S. Torres

**Affiliations:** 1 School of Public Health, University of Sao Paulo, 715 Dr Arnaldo Avenue, Sao Paulo, Sao Paulo 01246-904, Brazilgenirs@usp.br (G.R.S.); soares.maiara@usp.br (M.J.S.); 2 Department of Biochemistry, Memorial University of Newfoundland, St. John’s, NL A1B 3X9, Canada; fshahidi@mun.ca; 3 Departamento de Ciencias Vegetales, Facultad de Agronomía e Ingeniería Forestal, Pontificia Universidad, Católica de Chile, Casilla 306-22, Santiago, Chile

**Keywords:** *Paullinia cupana*, polyphenols, proanthocyanidins, alpha-glucosidase, bioactive compounds, type 2 diabetes

## Abstract

The Brazilian Food Supplement Law recently recognized that guarana (*Paullinia cupana*) contains bioactive substances, hence supporting its role as a functional food ingredient. The health benefits of guarana are associated, at least in part, to its phenolic compounds. However, to the best of our knowledge, there is no literature addressing the presence of phenolic compounds in the fraction containing insoluble-bound compounds and its contribution in terms of alpha-glucosidase inhibition. The concentration of phenolic extracts released from the insoluble-bound fraction required to inhibit 50% of alpha-glucosidase (IC_50_) activity was 5.8-fold lower than that present in the soluble counterpart. Both fractions exhibited a mixed inhibition mode. Fourteen proanthocyanidins (dimers to tetramers) present in the insoluble-bound fraction were tentatively identified by MALDi-TOF-MS. Future studies aiming at increasing the concentration of the soluble counterpart are deemed necessary. The results presented here enhance the phenolic database of guarana and have a practical impact on the procurement of nutraceuticals and functional ingredients related to the prevention and/or management of type 2 diabetes. The Brazilian normative on food supplements has been recently revised. This study lends support to the future inclusion of guarana powder in the list of sources of proanthocyanidins for the industry of food supplements.

## 1. Introduction

Guarana (*Paullinia cupana*) is a plant deeply rooted in the cultural identity of the Brazilian indigenous population, to whom it is credited the domestication and traditional processing of the plant’s fruit. The guarana-based beverage was consumed as an energy source for hunters and during fishing, and for the treatment of headache and fever [1]. Nowadays, this product of the Amazonian rainforest is known worldwide for its stimulating properties and potential health benefits. Studies have shown health effects related to guarana consumption, such as increased cognitive performance, improved energetic metabolism, lower prevalence of noncommunicable diseases, and improvement of oxidative stress markers [2,3,4,5]. These health effects have mainly been associated with the presence of caffeine and flavanols in guarana’s composition.

The studies of guarana have been focused on the polyphenols extracted with hydro-organic solvents as the representative of the polyphenol content. This fraction is recognized as the soluble polyphenols (SPP). However, the residue generated may still contain a significant amount of bioactive compounds. Insoluble-bound polyphenols (IBPP) are bound to the cell walls of the plant material. The analysis of the contribution of IBPPs to the total phenolic content of selected fruits and vegetables resulted in a mean of 57%, demonstrating its importance and high variability among vegetable sources [6]. The contribution of insoluble-bound phenolics depends on several aspects such as the nature of tested material (e.g., fruits, vegetables, cereals, legumes, and processing by-products) as well as the portion being analyzed (e.g., flesh, skin, leaf, stem, seeds). IBPP made a minor contribution to the total phenolic and total flavonoid contents of all six cranberry genotypes evaluated by Abeywickrama et al. [7]. IBPP from potato peel rendered as many total phenolics as the SPP fraction (free plus esterified phenolics). In contrast, IBPP from potato flesh did not contribute significantly to the total phenolic content of it [8]. Similarly, the content of IBPP in date seeds and leaves were negligible [9]. Wheat, rice, and some other cereals are good sources of IBPP while SPP was the most prominent fraction in millets [10,11,12,13]. The presence of IBPP was reported for peanuts, beans, lentils, and soybeans, among other legumes [14,15,16,17]. Likewise, IBPP were investigated in various plant food by-products [18].

Although a higher total phenolic content may suggest a higher potential inhibition against alpha-glucosidase, increasing evidence indicates that structural features of specific molecules involved may more appropriately explain the inhibition as discussed in detail elsewhere [19]. In short, it might be concluded that phenolic acids show lower inhibitory activity compared to flavonoids and that these are also influenced by the number of hydroxyl groups present in the molecules. In the case of proanthocyanidins (condensed tannins), polymeric structures show higher inhibitory activity compared to their monomeric counterparts (e.g., catechin and epicatechin). Due to their presence in the bound form in nature, the potential health effects of IBPPs are mainly related to gut health, with modulation of gut bacteria, protection of the colon mucosa, which may lead to systemic effects [20,21,22].

Another important strategy for harnessing the potential of the insoluble-bound polyphenols is increasing their bioavailability through auxiliary methods such as enzyme-assisted extraction, fermentation, and germination [23], which increases the ratio of the soluble to insoluble-bound phenolic compounds in a given sample. Other methods to improve the release of IBPP from the cell wall of the plant material include high-pressure- and ultrasound-assisted extraction [24,25]. The use of these methods has a positive impact on their potential health effects and opens new avenues in the development of functional foods and dietary supplements [26,27].

The Brazilian Food Supplement Law recently recognized that guarana contains bioactive substances, hence supporting its role as a functional food ingredient [28]. Both animal and human studies have found that the consumption of guarana powder was safe and caused no toxicity in small doses (30 mg/kg for animals and 200 mg/day for humans) [29,30]. While the composition of the soluble polyphenols from guarana is well established and reported [5,31,32,33], there is a clear gap in the available literature regarding the insoluble-bound fraction as a source of bioactive compounds. Considering that the guarana powder is traditionally consumed as a whole, as opposed to a water-based extract, the IBPPs are also ingested and may contribute to the overall health effects of guarana consumption. Moreover, as in other feedstocks [34], the residue generated from the production of guarana syrup might be explored for its insoluble-bound bioactive compounds.

The World Health Organization estimates that 422 million adults have diabetes worldwide, with an ascendant tendency brought by the increased incidence of type 2 diabetes [35]. Problems associated with diabetes include skin complications related to bacterial and fungal infections, ocular complications that may lead to blindness, and nerve damage, also known as diabetic neuropathy [16]. Furthermore, systemic inflammation and oxidative stress may also be involved [36,37]. The increasing intake of sweetened food and beverages elevates the risk of insulin resistance, which consequently decreases hepatic insulin sensitivity and triggers the onset of type 2 diabetes [38,39].

One of the strategies employed in the management of this metabolic disorder is the inhibition of carbohydrate-hydrolyzing enzymes, such as alpha-amylase and alpha-glucosidase, in order to control post-prandial glucose levels. In some countries such as Brazil and Argentina, among others, anti-hyperglycemics are provided by the government to the population free of charge in order to relieve the affected population from associated economic burden [40]. The side effects of acarbose, the most studied inhibitor of alpha-glucosidase, include abdominal distention, flatulence, meteorism, and, possibly, diarrhea [41]. In this way, bioactive compounds that might have an effect on the activity of these enzymes are of interest for human health promotion [42], with new techniques being developed for the discovery and screening of enzyme inhibitors found in herbs and foods [43].

Alpha-glucosidase is located in the brush border of the small intestine. It is well known that insoluble-bound phenolics have a negligible reactivity in this location, being more active after colonic fermentation. However, before investing time and financial resources in different methods (e.g., enzyme treatment and/or fermentation) to increase the concentration of soluble phenolics [26,27], which could provide a higher concentration of phenolic bioactives in the small intestine, one needs to make sure that the phenolics released from the cell wall of the plant material do actually bind to alpha-glucosidase. Accordingly, the present study focused on the presence of insoluble-bound polyphenols in guarana powder and evaluated its inhibition of the carbohydrate-hydrolyzing enzymes. The identification of the phenolic compounds was carried out by MALDi-TOF-MS. In short, our contribution may have an impact on the procurement of nutraceuticals and functional ingredients for the management and/or prevention of type 2 diabetes. The fraction containing soluble phenolics was also evaluated for comparative purposes.

## 2. Results and Discussion

### 2.1. Screening

In general, screening methods have low specificity, which may lead to over- or underestimation of the real values, but they have the advantage of being well-established and allow for the comparison of different samples. The total phenolic content (TPC) method is based on a redox reaction, and as such, it is dependent on the redox potential of the sample components. For polyphenols, their oxidation susceptibility is influenced by the degree of hydroxylation of the B-ring, the catechol group attached to the C2 position of the chromane ring and mainly associated with the antioxidant and metal chelating properties of polyphenols -, along with the presence of other reducing agents [44]. Certain sugars, amino acids, vitamin C, and other organic acids may influence the TPC values [45]. Due to the hydrolysis method, the IBPP fraction was further purified with solid-phase extraction (SPE) for the removal of interfering substances.

The TPC of the SPP and the IBPP fractions were 65.16 ± 0.19 mg and 3.97 ± 0.02 mg GAE/g of guarana powder (dw), respectively. The TPC values reported in the literature for the extractable polyphenols of guarana powder vary considerably and range from 8.4 to 151.8 mg GAE/g of the sample [5,46]. Different methods of extraction, as well as different origins of the guarana powder, explain the large variability of the results. In this way, the SPP fraction value is within the range expected for the sample. In contrast, there are no previous reports in the literature regarding the insoluble-bound polyphenol content of guarana powder, but similar investigations of green tea and dried persimmon resulted in the IBPPs contributing to 19 and 4% to the TPC, respectively [47,48], while the IBPP fraction of the guarana powder sample contributed 6% to the total value, although the hydrolyzation may impact the screening results. Different extraction methods, such as enzyme-assisted or fermentation, may be used in the future in order to release a higher proportion of the insoluble-bound polyphenols from the matrix [49].

### 2.2. Alpha-Glucosidase Inhibition Assay

**Figure 1:**
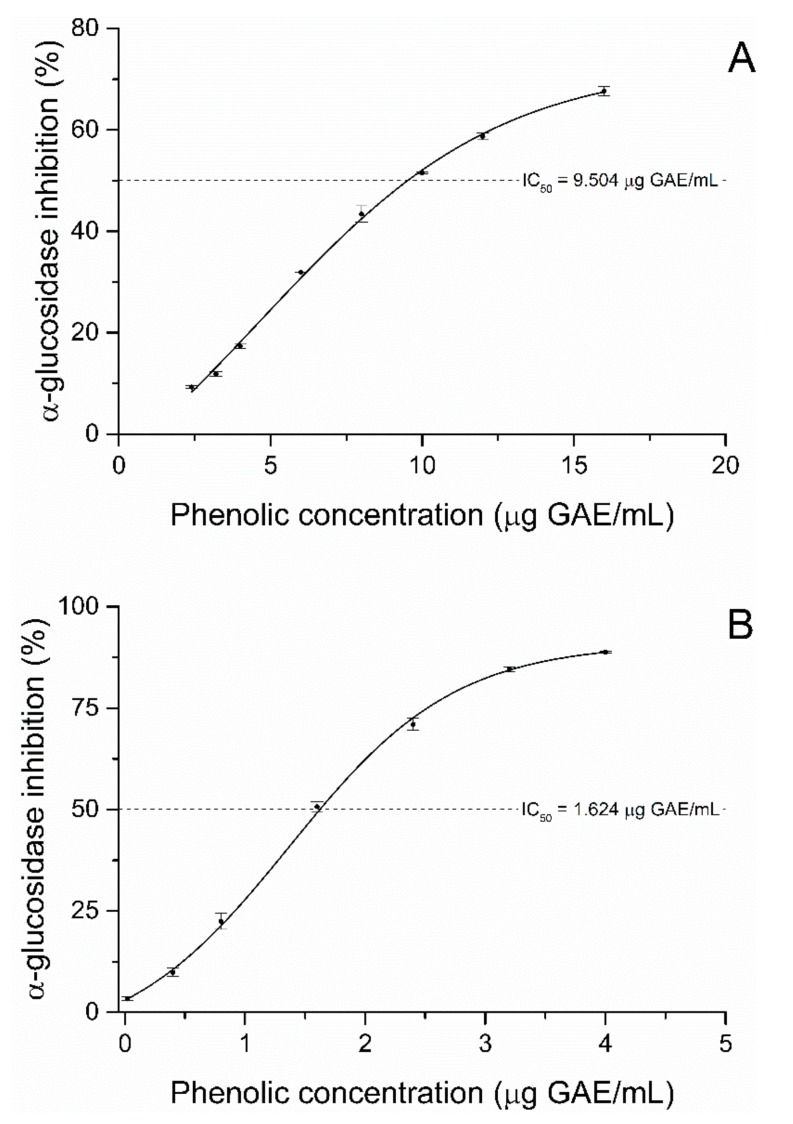
Dose-response curves of the inhibitory activity of (**A**) the soluble polyphenols (SPP) fraction and (**B**) the insoluble-bound polyphenols (IBPP) fraction on alpha-glucosidase.

The SPP and IBPP fractions inhibited the alpha-glucosidase activity in a dose-dependent manner. Their half-maximal inhibitory dose (IC_50_) was 9.50 and 1.624 µg GAE/mL, respectively (Figure 1). The efficacy of the IBPP fraction is also supported by its lower IC_50_ value compared to that of acarbose, which ranged from 36.0 to 107.3 µg/mL [50,51]. Green tea, oolong tea, and black tea had IC_50_ values of 10.02, 1.38, and 2.25 µg/mL, respectively [52,53]. Therefore, the values obtained for guarana powder are comparable to other well-established sources of polyphenols and even lower than that of acarbose, which is the most studied inhibitor of alpha-glucosidase.

The IC_50_ of SPP was approximately 6 times higher in comparison with the IBPP result. In a study with the soluble and insoluble-bound polyphenols of green tea, the inhibitory activity of the IBPP was 2.5 times higher than the SPP [48]. The difference in trend might stem from a distinction in the composition of the samples, which has a direct impact on the interactions with the enzyme.

### 2.3. Mode of Inhibition 

**Figure 2:**
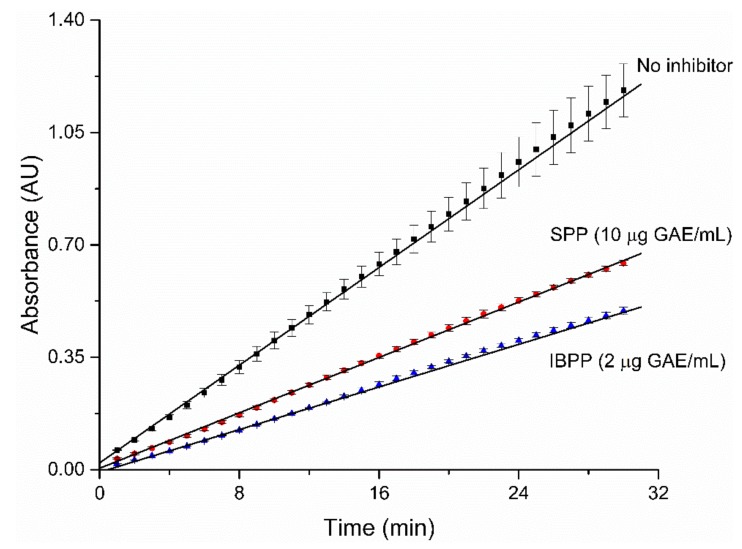
Reaction progress curve in the absence of inhibitors, and in the presence of SPP or IBPP as an inhibitor.

Enzyme inhibitors can be classified into two main classes: reversible and irreversible inhibitors. Irreversible inhibitors bind permanently to the enzyme and display steady-state velocities that approach zero, which can visually be assessed with a reaction progress curve. The progress curves for the alpha-glucosidase in the presence of SPP and IBPP are linear, indicating that they can be classified as reversible inhibitors (Figure 2) [54].

**Figure 3:**
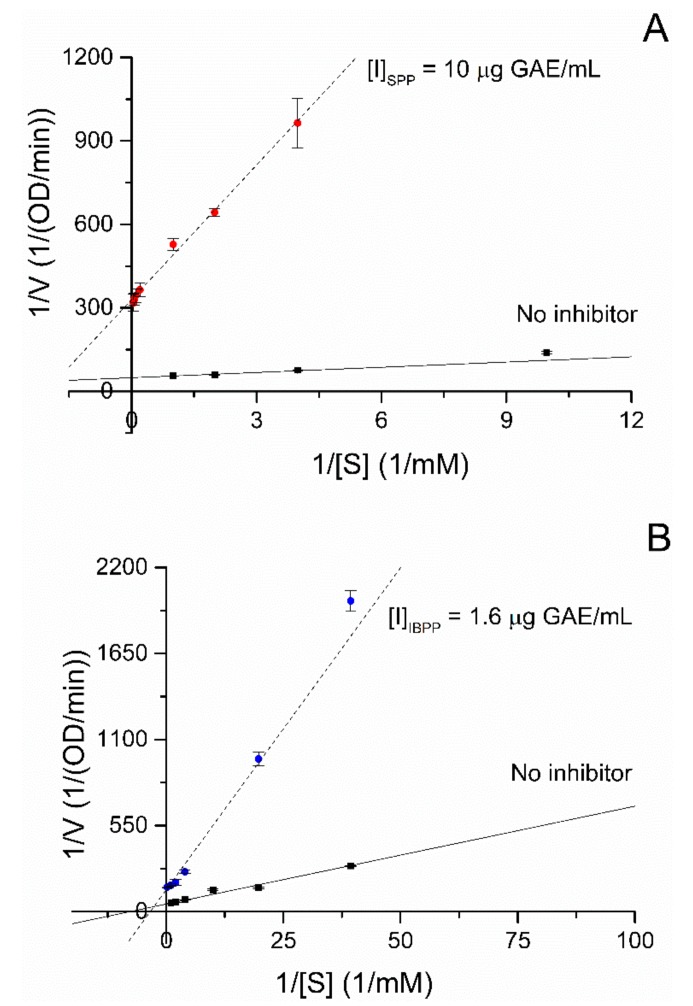
Enzyme kinetics analysis of both polyphenol fractions on alpha-glucosidase. (**A**) Lineweaver–Burk plot of the SPP fraction. (**B**) Lineweaver–Burk plot of the IBPP fraction.

Reversible inhibitors can be competitive, non-competitive, or mixed inhibitors based on how they interact with the enzyme, and these interactions have a direct impact on the values of the Michaelis constant (K_m_) and the maximum rate (V_max_). Competitive inhibitors compete directly with the substrate to bind in the enzyme’s active site, while non-competitive inhibitors bind to the enzyme-substrate complex outside of the active site. Lastly, mixed inhibitors bind to both the enzyme and the complex enzyme-substrate, outside of the active site of the enzyme. Consequently, in the presence of a mixed inhibitor, the values of K_m_ and V_max_ are altered in relation to the equilibrium constants, K_I_ and K’_I_. The Lineweaver–Burk plot offers a useful visual aid to identify the mode of inhibition for both the SPP and IBPP, the data lines had a different slope and a different intercept in comparison to the control plot, and the lines intersected in the second quadrant of the graph (Figure 3). In this way, SPP and IBPP can be classified as mixed reversible inhibitors for the alpha-glucosidase, and the Lineweaver–Burk linear equation can be written as follows [55]:

(1) $$\frac{1}{V_{0}}=\left(1+\frac{\left[I\right]}{K_{I}}\right)\times \frac{K_{m}}{V_{max}}\times \frac{1}{\left[S\right]}+\left(1+\frac{\left[I\right]}{K_{I}^{\prime }}\right)\times \frac{1}{V_{max}}$$

where [*I*] is the inhibitor concentration.

The inhibition kinetic parameters for alpha-glucosidase in the presence of SPP and IBPP were calculated based on Equation (1) and the appropriate mathematical relations. The equilibrium constants are expressed in Equation (2):

(2) $$K_{I}=\frac{\left[E\right]\left[I\right]}{\left[EI\right]}\;\;\;\;K_{I}^{\prime }=\frac{\left[ES\right]\left[I\right]}{\left[ESI\right]}$$

where [*E*] is the concentration of the enzyme, [*I*] is the concentration of the inhibitor, [*EI*] is the concentration of the complex enzyme-inhibitor, [*ES*] is the concentration of the complex enzyme-substrate, and [*ESI*] is the concentration of the complex enzyme-substrate-inhibitor. The inhibition kinetic parameters are presented in Table 1:

Equilibrium constants higher than 1 suggest that there are fewer inhibitors bound to the enzyme than in its free form, while constants lower than 1 suggest the opposite. In both cases, the K_I_ values were smaller than the K_I_’ values, indicating that SPP and IBPP bind more effectively to the free enzyme than to the enzyme-substrate complex. This difference is more pronounced in the SPP fraction, for which its K_I_’ value > 1 suggests that it binds poorly to the ES complex.

As evidenced by the enzymatic assay, the polyphenols of the guarana powder that are bound to the cell wall matrix have potential biological activities that remain unexplored. The interaction between phenolic compounds and proteins, which includes their capacity to inhibit enzymes, is related to their structure, size, number and position of substituents, and polyphenol complexity [56]. To the best of our knowledge, there are no published studies addressing the phenolic composition of the insoluble-bound polyphenols of guarana.

### 2.4. Insoluble-Bound Phenolic Profile

The soluble phenolic profile of guarana powder is well known and reported in the literature [5,31,32,33]. The main components are catechin and its isomer, epicatechin, and proanthocyanidin dimers.

Interactions between phenolic compounds and polysaccharides result in a major component in the occurrence of insoluble-bound polyphenols. When the cell wall is ruptured, as occurring during the grinding of the guarana seed to produce the guarana powder, polysaccharides and polyphenols interact rapidly non-covalently via a combination of hydrogen bonds and hydrophobic interactions between the phenolic compounds and the pectic fraction of the cell wall. The guarana powder pectin fraction is formed mostly of a homogalacturonan chain, similar to the composition found in apple pomace, which is another product recognized for its high proportion of insoluble polyphenols [57,58].

**Figure 4:**
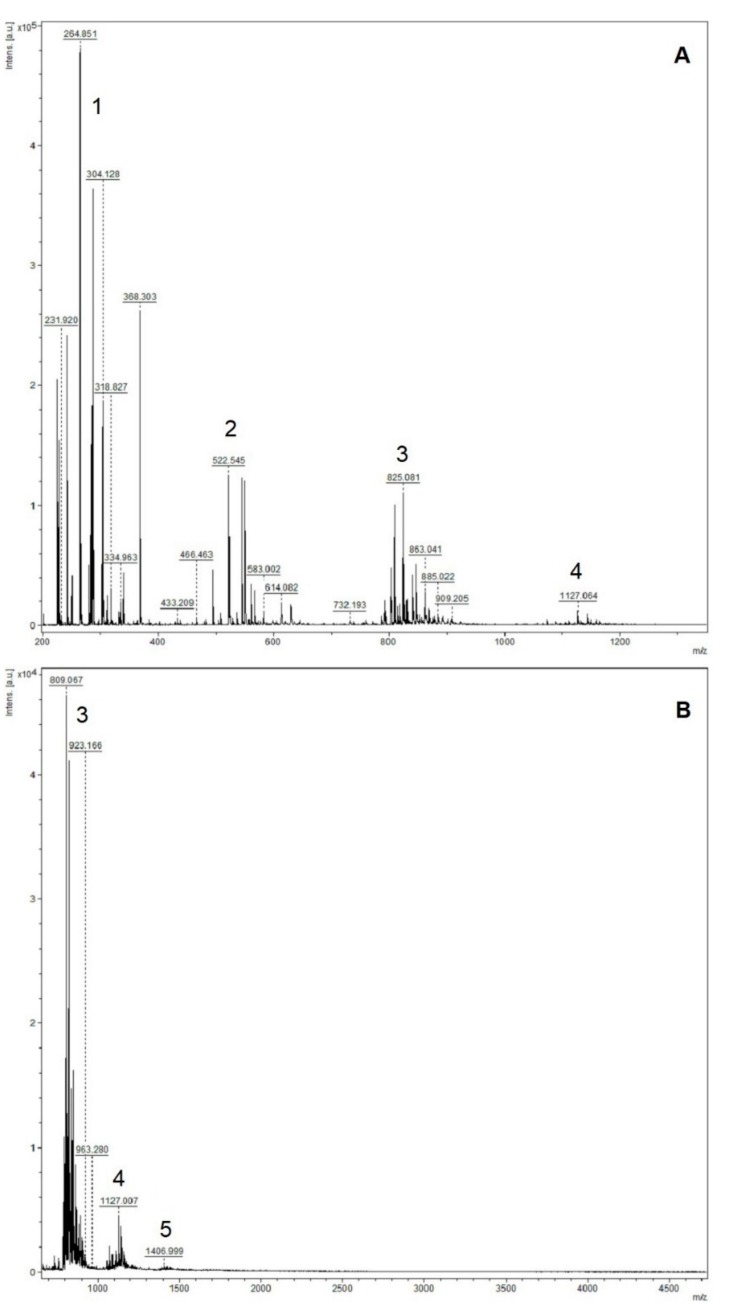
MALDI-TOF spectra of the insoluble-bound polyphenol from guarana powder. (**A**) 200–1400 Da. 1: Monomers peaks cluster; 2: Dimers peaks cluster; 3: Trimer peaks cluster; 4: Tetramer peaks cluster. (**B**) 700–4700 Da. 5: Pentamers peaks cluster.

The MALDI spectra of the insoluble-bound polyphenol fraction of guarana powder have the periodic clusters that are consistent with the analysis of polymeric/oligomeric structures, namely proanthocyanidins. The ion clusters are large, which indicates a mixture of types of oligomers (Figure 4). This complexity may be attributed in part to the alkaline hydrolysis, as it has been demonstrated that alkaline conditions are capable of cleaving the interflavan bond, and the presence of oxygen can cause further degradation. Additionally, the carbocation produced in the depolymerization process can react with other polyphenols in the solution, which may lead to the formation of hetero-oligomers and a higher proportion of dimers [59,60].

**Figure 5:**
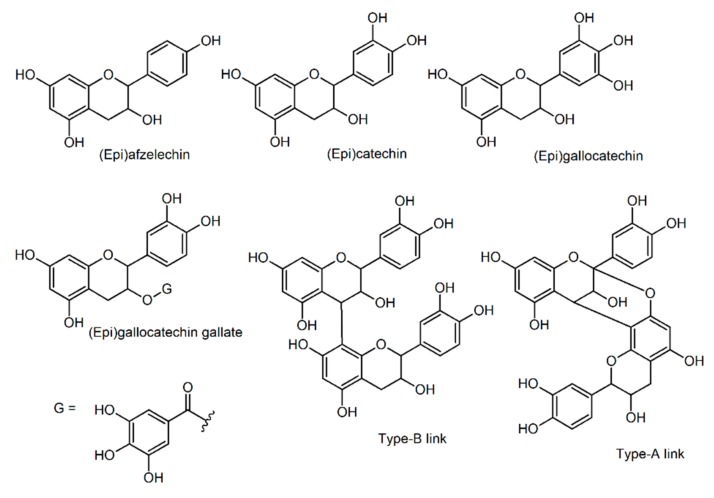
Simplified chemical structures of proanthocyanidin monomers and representation of the type-A and type-B linkages.

Plant-derived proanthocyanidin structures vary depending on the monomeric units, interflavan linkage type, hydroxylation pattern of the rings, and stereochemistry of the non-aromatic ring, complicating its characterization. The possible identities were calculated using the most common monomeric units of proanthocyanidins: (epi)afzelechin (MM: 274.084), (epi)catechin (MM: 290.079) and (epi)gallocatechin (MM: 306.074) (Figure 5). The insoluble-bound proanthocyanidins of guarana powder were detected as oligomers of low molecular weight (DP ≤ 5). However, larger oligomers may have their detection impaired by the saturation of the detector by the smaller compounds [61]. Therefore, their presence cannot be discarded. 

**Figure 6:**
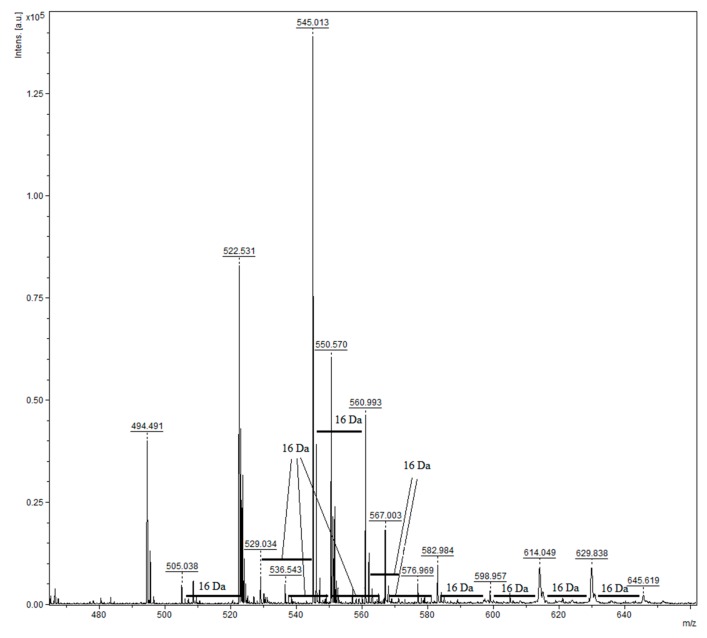
MALDI-TOF spectra of the insoluble-bound polyphenol from guarana powder: inset of the spectra highlighting the 16 Da increments associated with varying degrees of hydroxylation.

There are two distinct sets of compounds identifiable in the MALDI spectra. The first set corresponds to the proanthocyanidins composed of the most common monomers. Propelargonidins and procyanidins were detected, but no prodelphinidins were noticed. The peaks sets are comprised of series with varying degrees of hydroxylation, as evidenced by the 16 Da increments (Figure 6). These increments indicate the existence of hetero-oligomers, i.e., oligomers composed of a mixture of monomeric units. As expected due to the soluble polyphenol composition, galloylated compounds were not detected. Additionally, the majority of the interflavan linkage identified was of type-A [62,63,64]. The IBPP fraction composition is summarized in Table 2: 

The second set has a periodicity of 280 Da, which does not correspond to a known flavan-3-ol compound. The periodic clusters indicate that this set is comprised of oligomeric structures, and the 16 Da increments are also evidence of different hydroxylation patterns within the set. Although the identities of the compounds cannot be elucidated, they may be modified flavan-3-ol structures caused by depolymerization processes and/or the laser treatment [65,66].

In the latest years, several studies reported the presence of proanthocyanidins in the insoluble-bound fraction released from the cell wall [67,68,69]. In this way, the phenolic profile indicating the presence of dimeric and oligomeric proanthocyanidins linked to the cell wall of the plant material is consistent with the literature. The interaction between proteins and polyphenols are facilitated with the presence of polyphenols of higher molecular weight [70], which may help understand why the insoluble-bound fraction was seemingly more effective in the inhibition of the alpha-glucosidase. The IBPP fraction of the guarana powder has several oligomers bearing varying degrees of hydroxylation, thus providing different bond and interaction sites for the formation of the polyphenol-enzyme complex. Additionally, although afzelechin has never been identified in the SPP fraction of guarana powder, it was found to be present in the IBPP fraction and a recent study has shown a positive relationship between the proportion of dimers as well as the proportion of afzelechin extension units in proanthocyanidin fractions and their alpha-glucosidase inhibition capacity [71]. This difference between the SPP and IBPP composition may be an important factor for their effectiveness as alpha-glucosidase inhibitors. Likewise, according to the literature, the main soluble phenolic compounds present in guarana powder are monomers and dimers: catechin, epicatechin, procyanidin B1 and procyanidin B2. The presence of oligomers (trimers and tetramers) in the most active fraction (IBPP) lends support to a previous report [19] in which the authors suggested that, considering condensed tannins, oligomeric structures (<10 monomeric units) exhibit higher inhibitory activity compared to monomeric flavonoids.

The revised Brazilian normative on food supplements has been recently discussed by de Camargo and Silva [72]. Proanthocyanidins were mentioned among a few other phenolic compounds. However, only cranberry powder was mentioned as a source of proanthocyanidins. Guarana powder is listed as a well-known source of caffeine. However, the present study lends support to the suggestion made by these authors that guarana must be included as a Brazilian source of proanthocyanidins.

## 3. Materials and Methods 

### 3.1. Materials

Guarana powder was purchased from a local market (São Paulo, Brazil). All solvents and reagents were of analytical grade or higher. Catechin, epicatechin, alpha-glucosidase (from *Saccharomyces cerevisiae*), and 4-nitrophenyl-α-d-glucopyranoside (pNPG) were purchased from Sigma-Aldrich (St. Louis, MO, USA).

### 3.2. Soluble Polyphenol (SPP) Fraction Obtention

Homogenized guarana powder samples (0.8 g) were suspended in 20 mL of distilled water, methanol, and acetone (20:56:24, *v*/*v*/*v*), followed by homogenization with Ultra-Turrax (IKA T18 Basic, Taquara, Rio de Janeiro, Brazil) at 14,000 rpm for 3 min and centrifugation at 18,000× *g* for 15 min (Centrifuge 3–18 K, Sigma Laborzentrifugen, Osterode, Germany) [73]. Four extraction cycles were completed successively, and the supernatants were pooled together after filtration to remove solid impurities. The final volume (100 mL) was adjusted with distilled water, and the extract was stored in an amber flask at 4 °C.

### 3.3. Insoluble-Bound Polyphenol (IBPP) Fraction Obtention

The solid residue from the hydro-organic extraction was lyophilized and homogenized prior to its utilization. Residue samples (50 mg) were combined with 2 mL of 2 M NaOH, and the mixture was flushed with N_2_. The ultrasound-assisted (37 kHz and 100 W) reaction was carried out at 60 °C for 15 min. The solution pH was neutralized with 4 M HCl and, subsequently 2 mL of acetone-distilled water-acetic acid (70:29.5:0.5, *v*/*v*/*v*) was added to the solution, followed by homogenization with Ultra-Turrax at 14,000 rpm for 1 min and centrifugation at 10,000× *g* for 10 min [60]. Three extraction cycles were completed successively, and the supernatants were pooled together after filtration to remove solid impurities. The final volume (10 mL) was adjusted with distilled water, and the extract was stored in an amber flask at 4 °C.

The extract was further purified with solid-phase extraction (SPE) for the removal of salts, sugars, and other possible interferents. Briefly, the sample was diluted in a 0.1% acetic acid solution (1:1, *v*/*v*), and then loaded into a previously activated and equilibrated OASIS SEP-PAK cartridge (3 cc, 500 mg). The cartridge was flushed with 0.1% acetic acid solution, and the sample was eluted with acidified methanol (0.1% acetic acid) at a rate of 1 mL/min [74]. The eluted sample was stored in an amber flask at −20 °C.

### 3.4. Total Phenolic Content (TPC)

The appropriately diluted SPP and IBPP fractions (120 µL) were added to a transparent polystyrene microplate, followed by 50 µL of a 20% Folin-Ciocalteu reagent solution. The mixture was briefly homogenized and incubated at room temperature for 3 min. After this period, 30 µL of sodium carbonate (200 g/L) was added to the solution, and the mixture was homogenized and incubated at 37 °C for 1 h [75]. The sample absorbance was measured at 765 nm with a Molecular devices spectrophotometer (SPECTRAmax, Sunnyvale, CA, USA). Gallic acid was the standard for the calibration curve. The results were expressed as milligram of gallic acid equivalents per gram of dry weight of guarana powder or residue (mg GAE/g dw).

### 3.5. Enzymatic Assays

The SPP and IBPP samples had their organic solvents eliminated in a centrifugal vacuum concentrator (CentriVap) and were re-dissolved in water prior to the enzymatic analysis. Phosphate buffer (0.1 M, pH 6.8) was used in the assay.

For alpha-glucosidase inhibition assay, 80 µL of the samples in different concentrations were added to a transparent polystyrene microplate, followed by 20 µL of 0.2 U/mL of alpha-glucosidase. The mixture was briefly homogenized and incubated at 37 °C for 5 min. After this period, 100 µL of 4 mM pNPG was added to the solution, and the mixture was homogenized and incubated at 37 °C for 30 min. The reaction was stopped by adding 100 µL of 4 mM sodium carbonate, and the formation of *p*-nitrophenol was measured at 405 nm with a Molecular Devices spectrophotometer (SPECTRAmax, Sunnyvale, CA, USA). The inhibition percentage was determined according to Equation (3). The half-maximal inhibitory concentration (IC_50_) was calculated with the construction of a dose-response curve and non-linear regression.

(3) $$Inhibition\;\left(\%\right)=\left[1-\frac{\left(A_{sample}-A_{blank}\right)}{A_{test}-A_{control}}\right]\times 100$$

where *A_sample_* is the absorbance of the reactive medium, *A_blank_* is the absorbance of the reactive medium excluding the enzyme, *A_test_* is the absorbance of the reactive medium excluding the sample, and *A_control_* is the absorbance of the reactive medium excluding the sample and the enzyme.

The inhibition mode was investigated similar to the previous assay, but using a wide range of pNPG concentration to reach enzyme saturation and keeping the concentration of the enzyme and the inhibitor (SPP and IBPP) constants. The 30-min reaction was monitored at 405 nm with a Molecular Devices spectrophotometer (SPECTRAmax) in the kinetic mode. The kinetic parameters were calculated with the construction of a curve representing the relation between initial velocity (V_0_) and substrate concentration ([S]), the linearization of Lineweaver–Burk (Equation (4)), and the appropriate mathematical relations [48,54,55].

(4) $$\frac{1}{V_{0}}=\left(\frac{K_{m}}{V_{max}}\right)\times \frac{1}{\left[S\right]}+\frac{1}{V_{max}}$$

where *K_m_* is the Michaelis constant, and *V_max_* is the maximum velocity.

### 3.6. Mass Spectroscopy Analysis of the IBPP Fraction

The phenolic profile of the IBPP fraction was analyzed by matrix-assisted laser desorption/ionization (MALDI-TOF-MS, MALDI UltrafleXtreme Bruker Daltonics, Billerica, MA, USA). The ionization source was an attenuated N_2_ laser beam, with a repetition rate of 1000 Hz and 1500 shots. 2,5-dihydroxybenzoic acid (DHB) was initially tested as a matrix, but the best quality spectra were obtained without the use of a matrix. The sample was diluted in methanol, deposited onto the target, and left to dry out at room temperature. The data was acquired in the positive reflector mode. To determine the possible identities of the peaks by comparison, the ion mass was calculated according to Equation (5):

(5) $${\left[M+H\right]}^{+}=\sum M_{m}+\left(152.011\times G\right)-\left[\left(DP-1\right)\times L\times 1.008\right]$$

where *M_m_* is the molecular mass of monomers, *G* is the number of esterified galloyl substituents, *DP* is the degree of polymerization, and *L* is the type of interflavan bond (type-A, *L* = 4; type-B, *L* = 2) [61].

### 3.7. Data Analysis

The results were expressed as mean ± standard deviation (*n* = 3). All the data analysis and calculations were performed using the software OriginPro (OriginLab, version 2016, Northampton, MA, USA) and Microsoft Excel. The statistical analysis (Tukey’s test, *p* < 0.05) was performed using the software Statistical Package for the Social Sciences (SPSS version 24.0, SPSS Inc., Armonk, NY, USA).

## 4. Conclusions

Guarana powder, which has been recently mentioned amongst the trends in food bioactives [76], includes a range of polyphenols that remain in the residue after the conventional extraction of soluble phenolics. Insoluble-bound polyphenols showed a higher efficacy (lower IC_50_) in inhibiting alpha-glucosidase compared to that of soluble phenolics. Fourteen proanthocyanidins (dimers to tetramers) were possibly identified in the fraction containing insoluble-bound phenolics by MALDI-TOF-MS, suggesting their role as alpha-glucosidase inhibitors. This was the first step in prospecting the potential bioactivity of the phenolics present in the insoluble-bound form in terms of alpha-glucosidase inhibition. However, to release a higher proportion of them from the cell wall matrix, possibly increasing the concentration of soluble phenolics in the small intestine, other processes (e.g., enzyme-assisted extraction and/or fermentation) should be employed. The results presented here may have an impact on the procurement of nutraceuticals and functional ingredients related to the prevention and/or management of type 2 diabetes.

## Figures and Tables

**Table 1:** Calculated inhibition kinetics parameters for the soluble (SPP) and the insoluble-bound polyphenol (IBPP) fractions from guarana powder (*Paullinia cupana*) on alpha-glucosidase.

	K_m_ (mM)	V_max_ (OD/min)	K_I_ (µg/mL)	K’_I_ (µg/mL)	K’_I_/K_I_	Inhibition Mode
No inhibitor	0.128	0.020	/	/	/	/
SPP	0.489	0.003	0.403	1.735	4.30	Mixed
IBPP	0.292	0.007	0.287	0.847	2.95	Mixed

**Table 2:** Insoluble-bound proanthocyanidin composition from guarana powder (*Paullinia cupana*) ^a^.

	Calculated [M + H]^+^	Observed [M + H]^+^	Possible Identity
*Dimers*	544.136	545.013	Type-A afzelechin dimer
	560.131	560.993	Type-A afzelechin/catechin dimer
	576.126	576.969	Type-A catechin dimer
*Trimers*	814.188	815.082	Type-A afzelechin trimer
	830.183	831.052	Type-A (2)afzelechin/catechin trimer
	846.178	847.021	Type-A afzelechin/(2)catechin
	862.173	862.995	Type-A catechin trimer
*Tetramers*	1090.288	1089.061	Type-B afzelechin tetramer
	1106.283	1105.044	Type-B (3)afzelechin/catechin tetramer
	1122.278	1121.009	Type-B (2)afzelechin/(2)catechin tetramer
	1116.230	1117.052	Type-A (2)afzelechin/(2)catechin tetramer
	1132.225	1133.023	Type-A afzelechin/(3)catechin tetramer
	1148.220	1148.998	Type-A catechin tetramer
	1164.215	1164.962	Type-A (3)catechin/gallocatechin tetramer

a: Selected peaks of the IBPP fraction obtained with MALDI-TOF.

## Abbreviations

SPP	Soluble polyphenol
IBPP	Insoluble-bound polyphenol
MALDI	Matrix-assisted Laser Desorpsion/Ionization
TPC	Total Phenolic Content
